# Loss of Individual Mitochondrial Ribonuclease P Complex Proteins Differentially Affects Mitochondrial tRNA Processing In Vivo

**DOI:** 10.3390/ijms22116066

**Published:** 2021-06-04

**Authors:** Maithili Saoji, Aditya Sen, Rachel T. Cox

**Affiliations:** 1 Department of Biochemistry and Molecular Biology, Uniformed Services University, Bethesda, MD 20814, USA; maithilisaoji@gmail.com (M.S.); Aditya.sen@usuhs.edu (A.S.); 2 Henry M. Jackson Foundation, Bethesda, MD 20817, USA; 3 Alector Inc., 131 Oyster Point Blvd, San Francisco, CA 94080, USA

**Keywords:** mtRNase P, mitochondria, tRNA, Drosophila, mtDNA

## Abstract

Over a thousand nucleus-encoded mitochondrial proteins are imported from the cytoplasm; however, mitochondrial (mt) DNA encodes for a small number of critical proteins and the entire suite of mt:tRNAs responsible for translating these proteins. Mitochondrial RNase P (mtRNase P) is a three-protein complex responsible for cleaving and processing the 5′-end of mt:tRNAs. Mutations in any of the three proteins can cause mitochondrial disease, as well as mutations in mitochondrial DNA. Great strides have been made in understanding the enzymology of mtRNase P; however, how the loss of each protein causes mitochondrial dysfunction and abnormal mt:tRNA processing in vivo has not been examined in detail. Here, we used *Drosophila* genetics to selectively remove each member of the complex in order to assess their specific contributions to mt:tRNA cleavage. Using this powerful model, we find differential effects on cleavage depending on which complex member is lost and which mt:tRNA is being processed. These data revealed in vivo subtleties of mtRNase P function that could improve understanding of human diseases.

## 1. Introduction

The mitochondrial genome in metazoans is highly conserved, encoding the same 13 oxidative phosphorylation proteins, 2 ribosomal RNAs (mt:rRNA), and a complete set of 22 transfer RNAs (mt:tRNAs) required for translation [1]. Similar to its bacterial ancestor, mitochondrial DNA is transcribed as polycistronic RNA, which is cleaved into individual products post-transcriptionally [2]. In the metazoan mitochondrial genome, mRNAs and rRNAs are interspersed by tRNAs, an organization that is commonly referred to as the tRNA punctuation model [3]. With this arrangement, correctly processed mt:tRNAs release the other mtRNA products through the action of mitochondrial (mt) RNase P and RNase Z (ELAC2) acting at mt:tRNA 5′- and 3′-ends, respectively [4,5].

**Figure 1:**
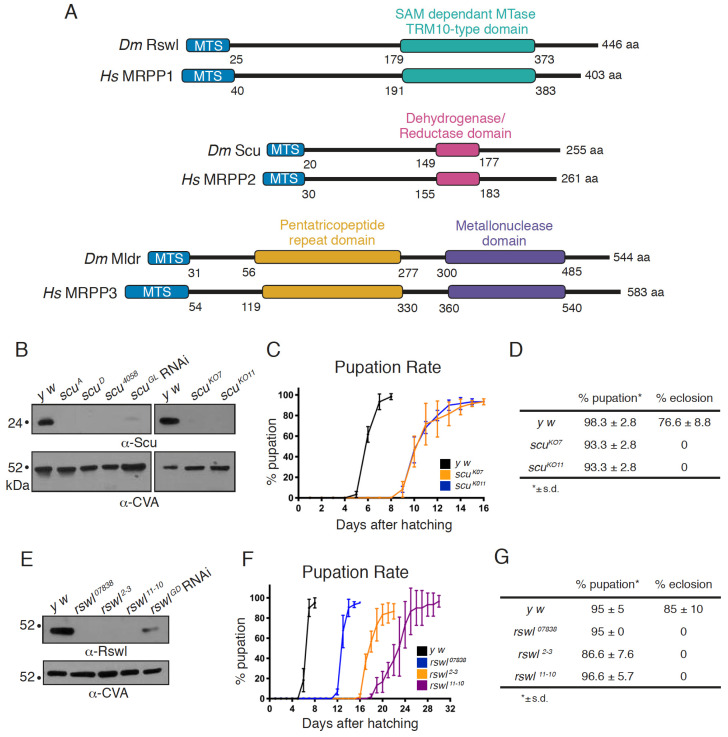
Loss of function alleles of *rswl* and *scu* cause lethality in flies. (**A**) Schematic showing the domain structure of Roswell, Scully, and Mulder and their homology in humans. The mitochondrial targeting sequence (MTS, blue) was predicted using MitoProt server, and the domain boundaries were predicted using Clustal Omega based on human homologs [30,31]. MTase: methyltransferase. (**B**–**D**) CRISPR/Cas9 induced loss of *scu* caused lethality. (**B**) Antibodies raised against Scu indicate all five *scu* alleles appear protein null via Western blot compared to the wild-type (*y w*), including ubiquitous *scu^GL^* RNAi expression. (**C**,**D**) *scu^KO7^* and *scu^KO11^* had delayed pupation (**C**) and failed to eclose (**D**) at room temperature. (**E**–**G**) The three *rswl* alleles did not have detectable protein on Western blot. Ubiquitous *rswl^GD^* RNAi expression shows reduced protein levels (**E**). (**F**) *rswl* mutant larvae eventually pupate but had delayed development, and none enclosed at room temperature (**G**). CVA: anti-ATP synthase. s.d. was calculated using GraphPad PRISM (**C**,**D**,**F**,**G**).

In the nucleus, canonical RNase P is a ribonucleoprotein with a catalytically active H1 RNA responsible for cleaving the 5′ leader sequence from precursor tRNAs to generate tRNAs with mature 5′-ends [6,7]. In contrast, in mitochondria, human mtRNase P carries out this function. mtRNase P is a three-protein complex comprised of mitochondrial ribonuclease P proteins (MRPP) 1, 2, and 3 (Figure 1A) [4]. MRPP1 (TRMT10C) is a methyltransferase that catalyzes the N^1^ methylation of adenosine or guanosine at the nineth position of 19 out of 22 human mt:tRNA [8,9]. MRPP2 (HSD17B10/SDR5C1) is a multifunctional protein of the small chain dehydrogenase reductase (SDR) family of proteins involved in amino acid catabolism and lipid metabolism [10,11]. MRPP3, a homolog of the single subunit PRORPs present in the nucleus and organelles of land plants, is the catalytic metallo-endonuclease that cleaves mt:tRNAs at the 5′-end [4,12,13,14]. MRPP1 and MRPP2 form a tight subcomplex which is required for the methyltransferase activity and stability of MRPP1 but not for the dehydrogenase activity of MRPP2 [4,9,15]. Methylation of mt:tRNAs by the subcomplex is not a prerequisite for the endonuclease activity of MRPP3 [9]. In vitro reconstitution experiments indicate that the MRPP1/2 subcomplex may function as a so-called “maturation platform” by enhancing the 3′-end cleavage efficiency of RNase Z and 3′-CCA addition [16]. For 5′-end processing, the MRPP1/2 subcomplex is thought to act as a scaffold and is obligatory for the catalytic activity of MRPP3 perhaps by enhancing Mg^2+^ binding and/or stabilizing the active site conformation of the endonuclease [4,9,16]. In addition, recent findings suggest that MRPP1/2 increases the affinity of MRPP3 for its pre-tRNA substrate and that the presence of MRPP3 can enhance the methylation activity of MRPP1/2 [17]. There is increasing evidence that 5′-end mt:tRNAs are not processed uniformly in vitro and in vivo by mtRNase P, which suggests that there are as yet unidentified additional factors or mechanisms that aid cleavage [18,19].

Defective mt:tRNA processing is associated with mitochondrial diseases in humans (reviewed in [20]). These diseases can be due to mutations in the mt:tRNA or in the proteins involved in mt:tRNA processing and maturation. There are approximately 275 known disease-associated mutations in mt:tRNAs, most of which are present outside the anticodon region [21]. Point mutations affecting the 5′- and 3′-end processing of mt:tRNAs are associated with maternally inherited hypertension, cardiomyopathies, ophthalmoplegia, and mitochondrial encephalomyopathy with lactic acidosis and stroke-like episodes (MELAS), to name a few [20,21]. Interestingly, different mutations in the same mt:tRNA often manifest diverse clinical symptoms, and there are no cures for these diseases. Mutations in the mtRNase P complex and RNase Z are linked to severe, sometimes fatal mitochondrial diseases. MRPP2 has the highest number of identified mutations that cause HSD10 disease [22]. HSD10 patients experience a variable age of onset and severity of the disease depending on the mutation. The patients display an array of classic multisystemic mitochondrial disease symptoms such as loss of cognitive and motor function, epilepsy, blindness, cardiomyopathy, and neurodegeneration [22]. Data collected from patient fibroblasts and in vitro experiments have shown that pathogenic mutations in MRPP2 affect tetramerization and dehydrogenase and/or mtRNase P activity [23,24,25]. Patients identified with pathogenic mutations in MRPP1 suffer from lactic acidosis, hypotonia, feeding difficulties, and deafness, and die as infants [26]. These patient fibroblasts showed an increased accumulation of unprocessed precursor mt:tRNAs but the methyl transferase activity was normal suggesting the in vivo role of MRPP1 in mtRNase P function is primarily responsible for the disease symptoms rather than its methyltransferase activity. A preprint has identified a mutation in MRPP3 that causes Perrault syndrome, a syndrome leading to neurological symptoms, deafness and primary ovarian insufficiency often caused by mutations in multiple proteins involved in mtDNA transcription and translation [27]. These patients apparently also accumulate unprocessed mtRNAs. In addition to patient data, there are mouse models that have been used to study the effects of loss of mtRNase P function. Mouse full-body knockouts of MRPP2 and MRPP3 are embryonic lethal [19,28]. Conditional knockouts of MRPP3 in heart and skeletal muscle lead to lethality at 11 weeks with the mice exhibiting cardiomyopathy and muscle defects [19]. The affected tissues showed increased accumulation of unprocessed RNA transcript, defective transcription, translation and mitoribosomal assembly. Mouse conditional knockout of MRPP2 in endothelial cells and immune cells showed lethality by 25 and 26 weeks, respectively, and had mitochondrial defects [28]. 

Since mitochondrial disease presentation is complex and multisystemic, model systems are a useful tool to dissect the in vivo role of MRPP1, 2, and 3 in mt:tRNA processing during development. We previously identified the *Drosophila* homologs of MRPP1 (Roswell (Rswl)) and MRPP2 (Scully (Scu)) and MRPP3 (Mulder (Mldr)) (Figure 1A) [29]. In *Drosophila*, all three proteins associate with each other and localize to mitochondria [29]. 

Mutations in *scu* and *mldr* and constitutive knockdown of *rswl*, *scu*, and *mldr* lead to delayed pupation and pupal lethality. The mutant larvae had defective mitochondria, marked by the loss of outer mitochondrial membrane permeability and reduced ATP levels [29]. *mldr* and *scu* mutants and *rswl* RNA interference (RNAi) showed an accumulation of precursor RNA transcripts on Northern blots probed with tRNAs located in different positional contexts within polycistronic transcripts [29]. Finally, we showed that overexpression of Rswl and Mldr caused lethality in flies and flies overexpressing Mldr, but not Rswl or Scu, had associated mitochondrial morphology defects [29]. 

In the current study, we identified null alleles of *rswl* and *scu* and used qPCR to examine how the loss of individual complex members affects mt:tRNA junction processing. We first develop CRISPR/Cas9 knockouts of *rswl* and *scu*. *rswl^2−3^*, *rswl^11−10^* and the previously uncharacterized P-element insertion mutation *rswl^07838^*, all showed delayed development and pupal lethality. *scu^KO7^* and *scu^KO11^* are also developmentally delayed and pupal lethal. These *rswl* and *scu* mutants were associated with mitochondrial morphology defects and reduced ATP production. Lastly, we examined mt:tRNA 5-′ and 3′-end processing of specific junctions located in different polycistronic transcript contexts in the absence of each protein. Overall, our qPCR data suggests that mutations in different mtRNase P complex members differentially affect junction processing for different mt:tRNA junctions. Loss of Rswl consistently had the greatest effect, and loss of Scu did not always affect junction processing depending on the mt:tRNA. Using the *Drosophila* model, this is the first time loss of all three mtRNase P members has been examined simultaneously in vivo for their effect on junction specific mtRNA processing.

## 2. Results

### 2.1. Protein Null Alleles of Scully and Roswell Confer Lethality and Mitochondrial Damage

**Figure 2:**
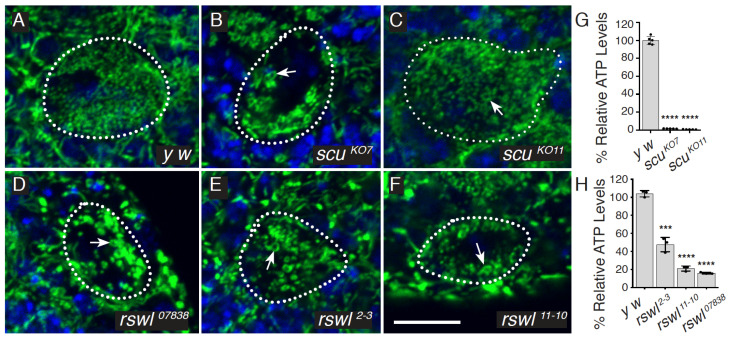
Loss of function of *rswl* and *scu* are associated with mitochondrial defects. (**A**–**F**) Neuroblast (NB) stem cells (dotted outlines) from larval brains labeled for mitochondria (green) and DAPI (blue). Neuroblasts in wild-type (*y w*) brains had small, dispersed mitochondria (**A**). In contrast, mitochondria in the neuroblasts of *rswl* and *scu* mutants (**B**–**F**) were swollen and ring-shaped (arrows). Number of NB with swollen mitochondria = 1/56 (**A**), 71/80 (**B**), 29/30 (**C**), 19/19 (**D**), 15/17 (**E**), and 22/25 (**F**). (**G**,**H**) Total relative ATP levels were reduced in *scu* (**G**) and *rswl* (**H**) mutant larvae. Green: anti-ATP synthase; blue: DAPI. Scale bar: 10 mm in F for A–F. Error bars: S.E.M. calculated using GraphPad PRISM using an unpaired *t*-test, two-tailed. (**G**, **H**). **** *p* < 0.0001; *** *p* < 0.001.

We previously characterized loss of mtRNAse P and found the mutant flies are pupal lethal, have reduced ATP, and accumulate longer, unprocessed mtRNA [29]. However, the *scu* alleles (*scu^A^* and *scu^D^*) are ethyl methanesulfonate (EMS) induced mutations, and the *rswl* mutant phenotype was produced by a single available RNAi knockdown strain. In addition, the Northern blot analysis indicated larger unprocessed mtRNAs species but did not analyze specific mt:tRNA junction processing. To generate better genetic tools for *rswl* and *scu*, we used CRISPR/Cas9 to create knockout alleles and generated antibodies against both proteins to determine protein levels in the mutants. *scu* knockouts were made by crossing transgenic flies stably expressing a single guide RNA complementary to the 5′-end of the protein-coding region with a transgenic fly line stably expressing Cas9 in the germline under control of a germline-specific promoter nosGAL4 [32,33]. Two knockout alleles, *scu^KO7^* and *scu^KO11^*, were randomly selected from a pool of 10 positives. Sequencing of *scu^KO7^* and *scu^KO11^* confirmed the expected edit by the guide RNA, which was repaired by non-homologous end joining (NHEJ), leading to a frameshift mutation at amino acid position 9 and 10 in the mitochondrial targeting sequence and a premature stop codon at amino acid positions 74 and 29, respectively (Figure S1A). To confirm these alleles are protein nulls, we generated antibodies to Scu. *scu^KO7^* and *scu^KO11^*, as well as *scu^A^* and *scu^D^*, did not have detectable protein on Western blot and have reduced transcripts (Figure 1B and Figure S2A). Antibodies raised against Scu colocalize with ATP synthase in *Drosophila* ovarian follicles using indirect immunofluorescence imaging (Figure S3). Using Western blots, antibodies raised against Scu also recognized an appropriately sized band in mitochondrial extract derived from adult flies (Figure S3). Both CRISPR/Cas9 induced null alleles caused a developmental delay in pupation and a failure to eclose into adults (Figure 1C,D). In addition, larvae had reduced ATP and mitochondria with mitochondrial morphological defects, indicative of mitochondrial dysfunction (Figure 2A–C,G) [34,35]. This agrees with our previous characterization of three other alleles of *scu*: *scu^D^* (S163F, point mutation in a conserved residue), *scu^A^* (Q159Stop, point mutation resulting in a C-terminal truncation), and *scu^4058^* (frameshift at E205, leading to a stop codon 20 aa downstream) [29].

Our previous work showed ubiquitous *rswl* RNAi expression caused lethality, low ATP levels, and an accumulation of larger mtRNA species [29]. While consistent with our other data on *mldr* and *scu* mutants, we could not be sure the phenotype reflected a true loss-of-function mutant phenotype as it only represented one RNAi line, and we did not have an Rswl antibody. In addition, unlike *mldr* and *scu*, this knockdown did not show mitochondrial morphological defects. Thus, we used CRISPR/Cas9 to generate a mutant allele. While we used two guide RNAs injected into embryos, sequence confirmation of the positive clones revealed that both alleles, *rswl^2−3^* and *rswl^11−10^*, were created by NHEJ of the Cas9 cleavage by only the guide RNA directed to the 3′-end of the coding region. This NHEJ leads to eight and eleven nucleotide deletions, respectively, and frameshift mutations in the C terminal extension (Figure S1B). To determine the effect of these alleles on the amount of available protein, we generated Rswl antibodies. Antibodies raised against Rswl colocalize with antibodies to ATP synthase in *Drosophila* ovarian follicles using indirect immunofluorescence imaging (Figure S3). This colocalization was not as strong compared to Scu (Figure S3). Since Scu is a homotetramer, this may be because there is a higher abundance of the Scu protein compared to Rswl, or it could be due to the anti-Rswl antibody not being as efficient as the Scu antibody for immunofluorescence. Using Western blots, antibodies raised against Rswl also recognize an appropriately sized band in mitochondrial extract derived from adult flies (Figure S3). Both *rswl* mutations result in protein nulls as judged by Western blot (Figure 1E), and the resulting transcripts were reduced (Figure S2B). A third allele, *rswl^07838^*, caused by a transposable element inserted into the first intron, also appeared protein null and had reduced transcript levels (Figure 1E and Figure S2B). In contrast, the previously characterized RNAi line has residual protein (Figure 1E). *rswl^2−3^* and *rswl^11−10^* delayed pupation (Figure 1F). All three *rswl* mutants failed to enclose and had reduced levels of ATP, similar to our previous result with *rswl RNAi* (Figure 1G and Figure 2H) [29]. Finally, in contrast to our initial observation of *rswl* RNAi, all three alleles caused mitochondrial swelling in larval neuroblasts (Figure 2D–F). 

### 2.2. Loss of mtRNase P Complex Members Differentially Affects mt:tRNA 5′-End Processing In Vivo

**Figure 3:**
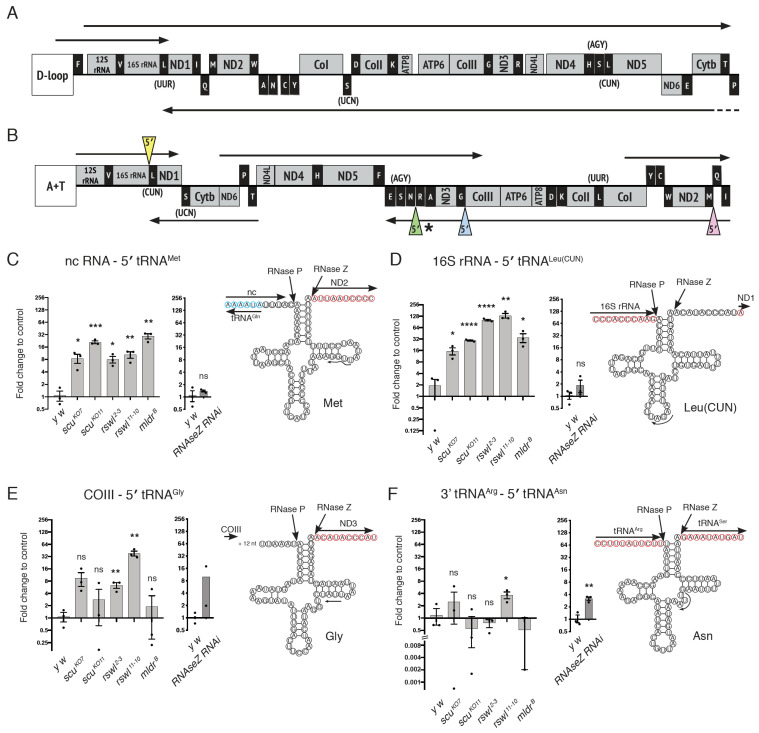
Mutations in mtRNase P complex proteins differentially affect mt:tRNA 5′-end processing. (**A**,**B**) Linear schematic of human (**A**) and *Drosophila* (**B**) circular mtDNA. Protein coding regions (mRNA) and ribosomal RNA (rRNA) are depicted in grey, whereas mt:tRNAs are in black. Both mtDNAs encode the same products in a slightly different order. Arrows indicate polycistronic transcripts, with humans containing three and *Drosophila*, five. The non-coding regions that control replication (human and *Drosophila*) and transcription (human) are the displacement (**D**) loop and the A+T region that is rich in adenine and thymine. The mt:tRNA 5′-ends tested are indicated with colored triangles (**B**). The asterisk indicates a junction that could also be recognized and cleaved at the mt:tRNA 3′-end. (**C**–**F**) Graphs representing the log_2_ fold change in qPCR levels and the corresponding predicted mt:tRNA cloverleaf structure for mt:tRNA 5′-end canonical junctions between (**C**, pink triangle) non-coding (nc) RNA: 5′ tRNA^Met^; (**D**, yellow triangle) 16S rRNA: 5′ tRNA^Leu(CUN)^; (**E**, blue triangle) COIII: 5′ tRNA^Gly^; (**F**, green triangle) 3′ tRNA^Arg^: 5′ tRNA^Asn^ for *scu*, *rswl, mldr*, and *UAS*-*RNaseZ* RNAi mutant larvae. The arrow within each tRNA diagram indicates the internal primer start site for qPCR analysis. The red circled nucleotides represent 5′ -> 3′ mtRNA, and the blue represents mt:tRNA encoded on the opposite strand. Bars on the graphs represent averaged 2^ddCt, and the y-axis is log_2_. Further explanation can be found in the materials and methods. The statistical significance was calculated relative to control (*y w*). Error bars: S.E.M. calculated using GraphPad PRISM using an unpaired *t*-test, one-tailed. * *p* < 0.05; ** *p* < 0.01; *** *p* < 0.001; **** *p* < 0.0001; ns: not significant.

*Drosophila* mtDNA is transcribed as five polycistronic transcripts compared to three for human mtDNA (Figure 3A,B) [36,37,38]. Both mtDNAs encode for the same products. In the polycistronic transcripts, mt:tRNA 5′-ends were found in different RNA contexts (i.e., next to rRNA, mRNA, tRNA, or non-coding RNA) (Figure 3A,B). It is possible the RNA environment could affect how mtRNase P recognizes cleavage sites. To analyze how the loss of any of the three mtRNase P components (Mldr, Rswl, and Scu) affects the processing of different mt:tRNA junctions, we collected first instar larval mutant extract and used qPCR to analyze mt:tRNA junction processing. Scu, Rswl, and Mldr are maternally loaded; thus, there is wild-type protein available early on in zygotically mutant embryos (Figure S4). However, Western blots probing extract isolated from mutant first instar larvae did not have detectable protein (Figure 1). In addition, we noticed that the mutant larvae appeared to grow somewhat asynchronously at later stages, particularly for the *rswl* mutants, and we wanted to ensure all the samples were taken at the same age. Therefore, we used an extract from first instar larvae for our analysis. qPCR was performed using primers that amplify directly across the junctions resulting in approximately 100-nucleotide products (Table S1). The PCR product amount was normalized within each sample against 18S RNA. To compare the amount of junction cleavage between genotypes, each junction reaction was compared to wild-type. If cleavage occurred at a normal rate, the mutant value would not be statistically different from the wild-type for a given set of primer pairs. Data are represented as fold changes on a log_2_ scale. 

We first examined 5′-end mt:tRNA junction processing for mt:tRNAs located adjacent to four different mtRNAs: non-coding (nc) mtRNA, mt:rRNA, mt:mRNA, and mt:tRNA. Three of the tested junctions have analogous junctions in human mtDNA (Figure 3B, pink, blue and yellow triangles), and the fourth is shown in a similar context (Figure 3B, green triangle). The prediction was that abolishing any mtRNase P complex member should result in decreased mt:tRNA 5′-end processing, and reduction of the 3′-end mt:tRNA processing enzyme RNase Z should have no effect. Two mt:tRNAs supported this prediction with qPCR. mt:tRNA^Met^ is downstream of nc mtRNA, and mutations in *scu*, *rswl*, and *mldr* caused a significant increase in the amount of unprocessed junction relative to wild-type (Figure 3C, pink triangle). We also examined the loss of RNase Z using the upstream activating sequence (UAS)/galactose metabolism (GAL)4 conditional expression system. Expressing *UAS-dRNaseZ^GD10927^* (RNase Z RNAi) ubiquitously using *daughterless GAL4* (*daGAL4*) resulted in a reduced *RNase Z* transcript (Figure S2D). In addition, knocking down RNase Z ubiquitously results in no pupation, as shown previously, with larvae only reaching second instar [39]. RNase Z RNAi has no effect on mt:tRNA^Met^ 5′-end processing (Figure 3C). mt:tRNA^Leu(CUN)^, which is directly downstream of mt:16S rRNA, has a similar pattern of unprocessed mt:tRNA (Figure 3D, yellow triangle). We also examined mt:tRNA^Gly^, which is adjacent to 18 non-coding nucleotides downstream of cytochrome c oxidase subunit III (COIII) (Figure 3E). Loss of both *rswl* alleles accumulated significantly more unprocessed junctions. In contrast, loss of *mldr* and *scu* null alleles did not. The final mt:tRNA 5′-end junction we tested, mt:tRNA^Asn^, was found in a cluster of five mt:tRNAs, and the 3′-end cleavage site of adjacent mt:tRNA^Arg^ was the 5′-end cleavage site of mt:tRNA^Asn^ (Figure 3F). For the 5′-end of mt:tRNA^Asn^, only the loss of one *rswl* null allele caused an accumulation of unprocessed junction. Loss of RNase Z also accumulated unprocessed junctions (Figure 3F).

### 2.3. mt:tRNA 3′-End and Non-Canonical Junction Processing Is Affected Differentially by Loss of mtRNase P Complex Members

**Figure 4:**
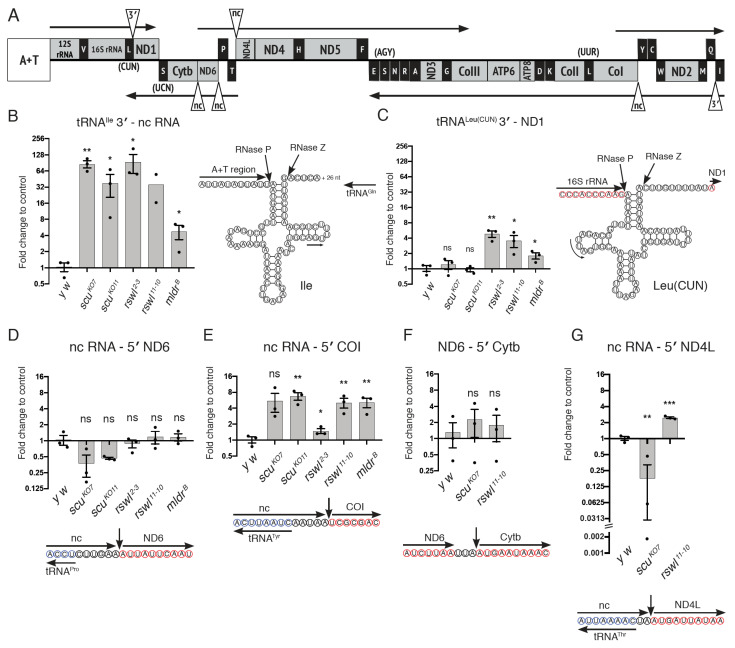
mt:tRNA 3′-end and non-canonical processing are differentially affected by mtRNase P. (**A**) Linear schematic of *Drosophila* circular mtDNA with triangles indicating the mt:tRNA 3′-end junctions and non-canonical junctions assayed. A+T indicates the adenine and thymine-rich non-coding region that controls replication. (**B**,**C**) Graphs represent the log_2_ fold change in qPCR levels in *scu*, *rswl*, and *mldr* mutant larvae for 3′-end junctions between tRNA^Ile^ 3′: non-coding (nc) RNA (**B**); tRNA^Leu(CUN)^ 3′: ND1 (**C**); ncRNA: 5′ ND6 (**D**); ncRNA: 5′ COI (**E**); ND6: 5′ Cytb (**F**); ncRNA: 5′ ND4L (**G**). The corresponding predicted cloverleaf structure of mt:tRNA (**B**,**C**) or nucleotide context of the 5′-end cleavage site (**D**–**G**). The internal mt:tRNA forward primer is indicated with an arrow (**B**,**C**). Red circled nucleotides represent 5′ -> 3′ mtRNA, and blue represent the mt:tRNA encoded on the opposite strand. Bars on the graphs represent averaged 2^ddCt, and the y-axis is log_2_. Further explanation can be found in the materials and methods. The statistical significance was calculated relative to control (*y w*). Error bars: S.E.M. calculated using GraphPad PRISM using an unpaired *t*-test, one-tailed. * *p* < 0.05; ** *p* < 0.01; *** *p* < 0.001; ns: not significant.

mt:tRNA 3′-end processing iwass achieved through the enzymatic activity of RNase Z/ELAC2. Data from cultured cells have shown that mt:tRNAs located on the heavy and light strands of human mtDNA have differential requirements for RNase Z/ELAC2 [40]. In addition, mouse tissues lacking MRPP3 appear to exhibit hierarchical processing for certain junctions where mt:tRNA 5′-ends must be cleaved before 3′-end processing can take place [19]. To determine the in vivo effect of loss of each RNase P complex member on 3′-end processing, we performed the same qPCR analysis on two mt:tRNA junctions (Figure 4A, triangles). mt:tRNA^Ile^ 3′-end is adjacent to nc mtRNA and is in an analogous position to human mt:tRNA^Pro^. Loss of all mtRNase P components caused an accumulation of unprocessed junction consistent with previous data (Figure 4B) [19]. The second junction we tested was the 3′-end of mt:tRNA^Leu(CUN)^, which is 10 nucleotides upstream of ND1 and is in an analogous position to human mt:tRNA^Leu(UUR)^. In contrast to mt:tRNA^Ile^, loss of *rswl* and *mldr* resulted in accumulation of product, but the loss of *scu* did not. 

In humans, all but two mtDNA products are flanked on at least one side by mt:tRNA. Thus, cleavage of mt:tRNA by mtRNase P (5′-end) or RNase Z/ELAC2 (3′-end) could conceivably initiate or complete processing of at least one end of most mtRNA products. However, there are certain non-canonical junctions in mtRNA that are not adjacent to mt:tRNA. How these junctions are cleaved is not completely understood. In *Drosophila*, we tested four out of the five non-canonical junctions (Figure 4A, triangles). We examined two junctions with all mutants (Figure 4D,E) and two more with a subset of mutants (Figure 4F,G). nc RNA-ND6 and nc RNA-cytochrome c oxidase subunit I (COI) are in a similar RNA context because while the upstream sequence is non-coding, it is mirror sequence for mt:tRNA^Pro^ and mt:tRNA^Tyr^, respectively, encoded on the opposite strand (Figure 4D,E, blue circles) [41]. Thus, the secondary structure derived from the mirror mt:tRNA could render positional information to the enzyme. Despite this similarity, a loss of mtRNase P had no effect on junction processing of nc RNA-ND6, but a loss of mtRNase P for all three proteins caused junction accumulation of nc RNA-COI (Figure 4D,E). While not all mutants were tested for ND6-cytochrome b (Cytb) and nc RNA-ND4L, these junctions also showed differential sensitivity (Figure 4F,G). Loss of *scu* and *rswl* had no effect on junction processing for ND6-Cytb. Only the loss of *rswl* led to an accumulation of unprocessed junction of nc RNA-ND4L. These data support that mtRNase P is required for certain non-canonical junction processing, but in *Drosophila*, this requirement was not equal for all mtRNase P subunits.

### 2.4. Loss of RNase P Differentially Affects the Abundance of Individual Transcripts Derived from the Same Polycistronic Transcript

**Figure 5:**
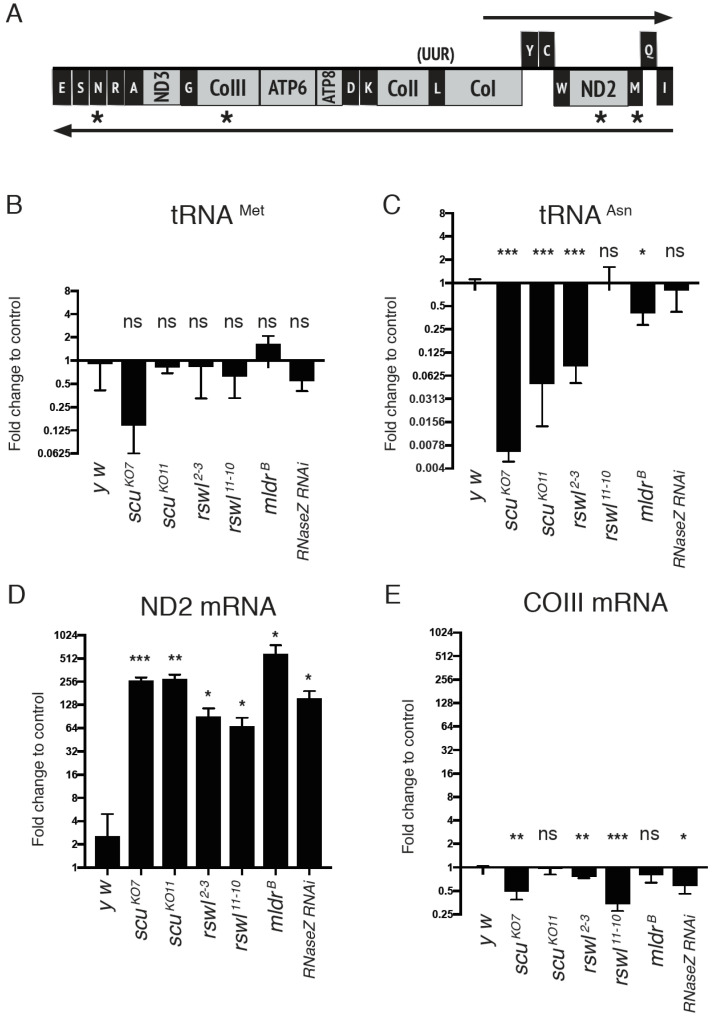
Steady state levels of mt:tRNAs and mt:mRNAs in mtRNase P mutants. (**A**) Graph of the analyzed mtRNA species (asterisks) located on the same polycistronic transcript. The graphs represent the log_2_ fold change in the steady-state amounts of mt:tRNA^Met^ (**B**), mt:tRNA^Asn^ (**C**), ND2 mRNA (**D**), and COIII mRNA (**E**) in *scu*, *rswl*, *mldr*, and *RNaseZ RNAi* mutants. Bars on the graphs represent averaged 2^ddCt, and the y-axis is log_2_. Further explanation can be found in the materials and methods. Error bars: S.E.M. calculated in GraphPad PRISM. *p*-values were calculated using GraphPad PRISM using an unpaired *t*-test, two-tailed, and compared each mutant to the *y w* control. * *p* < 0.05; ** *p* < 0.01; *** *p* < 0.001; ns: not significant.

In humans, deep sequencing has shown mtRNA levels vary even though they are located within the same polycistronic transcript [42]. In mice, mt:rRNAs and mt:mRNAs located on the heavy strand are differentially affected with loss of LRPPRC, a pentatricopeptide repeat (PPR) containing protein that regulates mtRNA stability [43]. mt:mRNAs are reduced in LRPPRC knockout mouse tissues, whereas the mt:rRNAs and the intervening mt:tRNA^Val^ are increased. We wanted to know if transcript abundance in the mtRNase P mutant background could potentially explain the increase in unprocessed junctions. Therefore, we measured four different transcripts in the same polycistronic transcript using qPCR from all of the mutants analyzed (Figure 5A, asterisks). The primers used spanned the single mtRNA, and thus, this analysis did not reflect whether the single mtRNA was present in a larger transcript. We found that mt:tRNA^Met^ levels in mtRNase P mutant backgrounds were not substantially increased compared to wild-type (*y w*) (Figure 5B) even though the mutants accumulated unprocessed junction (Figure 3C). mt:tRNA^Asn^ transcript levels were decreased in some mutant backgrounds and similar to wild-type in others (Figure 5C) while only the loss of *rswl* and *RNase Z* accumulated unprocessed junction (Figure 3F). We also measured ND2 and COIII using qPCR. These transcript levels were differentially affected by the genetic background, with loss of mtRNase P and RNase Z causing an accumulation of ND2 whereas COIII levels were reduced or unaffected (Figure 5D,E). Similarly, mt:tRNA^Asn^ and COIII transcripts showed no increases in the mutant backgrounds compared to wild-type as analyzed by Northern dot blots (Figure S5).

## 3. Discussion

### 3.1. Protein Null Mutants for In Vivo Analysis of All Three RNase P Complex Members

The human mitochondrial diseases caused by mutations in mtRNase P proteins are severe, mostly leading to infantile to juvenile lethality [22,26]. There are multiple mutations identified in each protein leading to disease symptoms, but the age of onset of the disease, the severity, and the symptoms vary between mutations in the same protein. Additionally, MRPP2 and MRPP1 are multifunctional proteins which makes teasing out the effect of mutations in vivo challenging. Most information on mt:tRNA processing has been collected from cultured patient fibroblasts and protein knockdown experiments in cultured cells. MRPP3 and MRPP2 conditional knockout mice are available, which has been particularly helpful with respect to characterizing MRPP3 function in vivo [19,28]. However, the effect of reducing all three proteins in the mtRNase P complex on specific mt:tRNA junction processing has not been systematically examined in vivo. In order to do this, we created and confirmed protein null alleles for *rswl*, *scu*, and *mldr*, allowing a comparative analysis of the effect of each protein on development and junction processing. 

### 3.2. Analyzing Specific mt:tRNA Junction Processing In Vivo

In vitro studies using purified proteins to test cleavage of a single exogenously added (pre)mt:tRNA have yielded much information on enzyme kinetics, the importance of the MRPP1/2 subcomplex and enzymatic activity requirements for methyltransferase (MRPP1) and endonuclease (MRPP3) activity, including the effect of pathogenic mutations on cleavage [18,44,45]. Additional experiments have used RNAi knockdown in cell culture lines, such as HeLa cells, or patient-derived cultured fibroblasts [4,5,26,46]. In vivo work has been performed in *Drosophila*, and for MRPP2 and 3, using a tissue-specific knockdown in mice [15,19,29]. The consistent conclusion from these studies is that the lack of any mtRNase P component causes the accumulation of larger mtRNA species. Using an MRPP3 heart-specific conditional knockout mouse model, Rackham et al. showed defects in mtRNA processing, and their data support a model by which 5′-end processing precedes 3′-end processing. However, the effect of a knockdown of each mtRNAse P protein on specific junction processing has not been examined in vivo. In addition, an advantage of in vivo analysis is the potential to help identify unknown mechanisms or co-factors that can potentially carry out mtRNA processing in the absence of mtRNase P. For example, S-adenosyl methionine (SAM) has recently been shown to act as a co-factor necessary for MRPP1 function in vitro but not for its methyltransferase activity [44]. Previously, we found loss of Rswl (MRPP1), Scu (MRPP2), and Mldr (MRPP3) caused an accumulation of larger mtRNA species [29]. To more precisely test the requirement of each protein specifically on 5′- or 3′-end junction processing, we designed primers for qPCR that spanned single junctions. This allowed us to directly measure processing of 5′- or 3′-end junctions in different mtRNA contexts with loss of Scu, Rswl, or Mldr. Many of the findings support previously published data; however, we found certain junctions are differentially affected by the loss of specific mtRNase P complex members in *Drosophila*.

MRPP1 is responsible for the methylation of m^1^G9 or m^1^A9 in mt:tRNAs. It is not known when methylation happens in vivo—before, during, or after cleavage. This RNA methylation can either block the action of reverse transcriptase or cause it to misincorporate a nucleotide. If this methylation blocks reverse transcription of RNA isolated from wild-type larvae, the resulting amount of cDNA across the 5′-end junction could be artificially decreased, thus enhancing the relative amount of unmethylated product present in the mutant extract. However, for this to be the case, it would assume that methylation precedes cleavage or that they are tightly coupled. In vitro data from the Rossmanith lab indicated that while mt:tRNA can be methylated by the holoenzyme, methylation, and cleavage are, in fact, uncoupled [9]. In addition, reconstituted mtRNase P with a methyltransferase-dead MRPP1 was still able to cleave (pre)mt:tRNA [9]. Processing is known to be highly efficient in wild-type conditions in vivo; thus, we would expect to capture very low levels of steady-state unprocessed mt:tRNA junctions in normal wild-type larvae [29]. The qPCR data could also be affected by differential mtRNA stability in wild-type versus mutant extract. Transcripts derived from the heavy strand of human mtDNA showed differential stability in the absence of LRPPRC, a protein that regulates mtRNA stability [43]. While we did see an effect on levels of ND2 (increased), mt:tRNA^Asn^, mt:tRNA^Met^, and COIII were either the same or decreased in mutant extract compared to wild-type. Importantly, differences in unprocessed junction accumulation for mt:tRNA^Asn^ and mt:tRNA^Met^ did not correlate with differences in total mt:tRNA levels (Figure 3 and Figure 5). Therefore, we believe our observed accumulation of unprocessed junctions using this method reflects the amount of mtRNA processing occurring in vivo. 

### 3.3. Canonical Junction Cleavage Is Most Affected by the Loss of Rswl

We tested four canonical mt:tRNA 5′-end sites for processing (Figure 3) with each junction in a different RNA context which could potentially affect cleavage site recognition. The preceding transcript sequence was either directly juxtaposed to the mtRNase P cleavage site (Figure 4D,F), or there was a larger (Figure 4C) or smaller (Figure 4E) number of non-coding nucleotides preceding the cleavage site. In addition, within the polycistronic transcript, there could be positional information from mt:tRNA folding within the forward sequence (Figure 4F, mt:tRNA^Arg^) or in mirror mt:tRNA (Figure 4C, mt:tRNA^Gln^) [41]. The MRPP1/2 subcomplex is thought to first bind mt:tRNA for correct positioning for subsequent cleavage by MRPP3 [16]. While the loss of Rswl, Scu, and Mldr resulted in an unprocessed junction for mt:tRNA^Met^ and mt:tRNA^Leu(CUN)^, only the loss of Rswl resulted in increased junction for mt:tRNA^Gly^, which was somewhat unexpected. If the 5′-end of mt:tRNA^Gly^ relies on mtRNAse P, one would predict missing any of the mtRNase P components would disrupt mtRNA transcript processing. In addition, the loss of Scu should have the same effect as the loss of Rswl since MRPP2 forms a tight subcomplex with MRPP1 [9] and is required for mt:tRNA methylation [47] and patient-derived HSDB10 fibroblasts and siRNA knockdown of MRPP2 leads to decreased MRPP1 protein [15]. The greater effect of Rswl loss on unprocessed junctions was consistent with in vitro experiments showing loss of MRPP1 caused the largest accumulation of unprocessed mtRNA junctions [5], although Sanchez et al. did not test MRPP2 knockdown. However, in contrast, the MRPP3 conditional mouse knockout caused accumulation of mt:tRNA^Gly^ 5′-ends based on parallel analyses of RNA ends (PARE) analysis [19] and also qPCR, although to a lesser degree than MRPP1 knockdown [5]. For this mt:tRNA junction, in vivo, Rswl may have additional methylation functions that, when lost, could cause unprocessed mtRNA accumulation through an unknown mechanism. Further enzymatic analysis of mtRNase P from larval extract will be required in the future to determine the mechanism.

### 3.4. Loss of Rswl Affects Processing within a Cluster of mt:tRNAs in Drosophila

tRNAs are believed to start building nascent secondary structures right after transcription due to energetically favorable hydrogen bonding [48]. *Drosophila* mtDNA encodes for a stretch of five mt:tRNAs and one mirror mt:tRNA that is closely juxtaposed (Figure 3B,F). This organization is similar to a cluster of four mt:tRNAs and one mirror mt:tRNA in human mtDNA (Figure 3A), although the mt:tRNAs encoded are not the same. Due to this arrangement, efficient cleavage at either the 5′- or 3′-end could remove the mt:tRNAs from the transcript (Figure 3F). In cultured HeLa cells, ELAC2 and MRPP1 RNAi caused a significant accumulation of mtRNA flanking ND2-tRNA^Ala^, although the specific primers used for the qPCR analysis are unclear [5]. In contrast, primers flanking the entire five-mt:tRNA cluster (ND2-COI) had a much smaller effect. In our analysis, loss of only one allele of *rswl* accumulated unprocessed junction for mt:tRNA^Asn^, the middle mt:tRNA (Figure 3B,F). In addition, RNase Z RNAi had a significant accumulation of product (Figure 4F), indicating the 3′-end cleavage of mt:tRNA^Arg^ could not compensate for the loss of *rswl*. These results are consistent with Sanchez et al. Loss of Scu had no effect, in a similar manner to mt:tRNA^Gly^ (Figure 3E). While the loss of Mldr trended towards normal, we were unable to get the primer pair working sufficiently well to be able to draw a conclusion. 

### 3.5. mt:tRNA Processing Is Not Always Dependent on Scu

Previous work examining an MRPP3 conditional knock-out mouse used PARE and RNAseq to investigate the degree to which loss of MRPP3 affected the presence of 5′-ends [19]. Rackham et al. found loss of 5′-end processing also reduced 3′-end processing gave rise to a hierarchical cleavage model whereby mtRNAse P activity is required before ELAC2 is able to cleave. This observation agrees with in vitro studies showing 3′-end cleavage by ELAC2 is enhanced in the presence of MRPP1/2 [16]. We examined 3′-end cleavage of mt:tRNA^Ile^ and found the loss of Rswl, Scu, and Mldr caused an increase in 3′-end unprocessed junctions in agreement with this model. We also examined mt:tRNA^Leu(CUN)^ (Figure 4C). The loss of Rswl and Mldr accumulated unprocessed junctions, also in agreement. However, as with our 5′-end analysis (Figure 3), the loss of Scu did not accumulate unprocessed junction. The primers for this junction were internal for mt:tRNA^Leu(CUN)^ and ND1, thus covering the 3′- and 5′-ends for each transcript, respectively (Table S1). Thus, loss of Scu also did not affect 5′-end cleavage of ND1. Again, this was surprising as the MRPP2 homotetramer is thought to form a highly stable subcomplex with MRPP1, but these results suggest in the absence of Scu, cleavage could still occur. While the organization of this region is the same between *Drosophila* and human, the human polycistronic transcript has only two nucleotides between the RNase Z cleavage site on mt:tRNA^Leu(CUN)^ and the start of ND1, whereas *Drosophila* has ten nucleotides. Perhaps this slightly larger gap changes enzyme kinetics between the two species.

We also examined several sites with junctions that are not predicted to be recognized by mtRNase P (non-canonical). Some of these junctions are unique to *Drosophila*, while some have a somewhat similar structure in humans. The 5′-end of ND6 was unaffected by the loss of Scu, Rswl, and Mldr. This is similar to Cytb processing in the MRPP3 conditional knockout mouse, which is located in a similar genomic organization [19]. In contrast, the 5′-end of COI was affected by the loss of all three proteins (Figure 4E) even though there is a mirror mt:tRNA^Tyr^ upstream, which is similar to 5′-end ND6. Finally, the two unique junctions, 5′-end Cytb (Figure 4F) and 5′-end ND4L (Figure 4G) appeared either unaffected or only affected by *rswl*, respectively, although we only tested one allele of each. The data for the non-canonical junctions support the notion that there are additional mechanisms for the cleavage of 5′-end junctions in some instances [19].

### 3.6. mtRNase P Action In Vivo

While mtRNAse P is clearly critical for 5′-end mt:tRNA processing, there is increasing support that other unidentified proteins or mechanisms may be involved in this process. Careful in vitro analysis has tested the requirement of the MRPP1/2 subcomplex and MRPP3 on 5′-end cleavage and methylation. However, this is the first time the individual proteins were removed in vivo and tested for their ability to process specific junctions. There is a rising consensus that Rswl/MRPP1 is the most critical protein, likely due to its strict requirement for MRPP3 cleavage and its enzymatic function as a methylase. Thus, it is not surprising junction processing is most sensitive to loss of Rswl. What is unusual is that Scu (Figure 3E,F and Figure 4C) and even occasionally Mldr (Figure 3E) appeared to be dispensable for certain junctions. One possibility is that there are additional mechanisms in vivo that can partially rescue the loss of mtRNase P. mtRNase P does not have an RNA component [4]. However, RNase P RNA activity has been copurified with mitochondria, and the RNA binds to PNPase to process mtRNA [49,50]. In addition, the nuclear DNA-encoded lncRNA mitochondrial RNA processing endoribonuclease (RMRP), which cleaves and processes rRNA in the nucleus, is in the mitochondrial matrix [51]. Thus, there are likely additional mechanisms that can compensate for the loss of one or more members of mtRNase P in vivo.

Mitochondrial diseases are pleiotropic, affecting multiple organs and systems. With only palliative care available, identifying the basic molecular mechanisms underlying these complex diseases is paramount to understanding disease etiology and progression. *Drosophila* has emerged as an excellent model for studying mitochondrial diseases that can be used to better understand how the loss of individual proteins affects the basic cellular biology underlying mitochondrial dysfunction [52,53]. Modeling mitochondrial diseases in *Drosophila* has the advantage of balancing a fast life cycle and well-developed genetics with somewhat complex organ systems that often have an analogous function to humans [54]. In humans, mutations in each of the mtRNase P subunits, as well as mtDNA mutations affecting the cleavage site, can cause loss of mt:tRNA cleavage and processing, which ultimately leads to mitochondrial disease. Using the strengths of *Drosophila* genetics, we have tested the loss of all three mtRNase P subunits in vivo and how this loss alters the cleavage of specific mt:tRNA junctions. By measuring discrete junctions, we found differential requirements of specific junctions for mtRNase P complex members and also apparent differences in processing which may be dependent on the flanking regions. These data start to tease apart the requirements for each protein and suggest there are additional mechanisms that can compensate for the loss of specific mtRNAse P components. Understanding the role of mtRNase P in *Drosophila* offers the opportunity to model diseases caused by aberrant mtRNase P function and could ultimately aid in further understanding of disease progression. 

## 4. Materials and Methods

### 4.1. Fly Stocks

The following stocks were obtained from Bloomington stock center: P(PZ)rswl^07838^ cn^1^/CyO; ry^506^, Ab(1)os^s^; y^1^ w* scu^4058^ upd1^os-s^ upd3^os-s^/FM6; y^1^ w* scu^A^ FRT19A/FM7i, ActGFP; y^1^ w* scu^D^ FRT19A/FM7i, ActGFP; y^1^ w* mldr^B^ FRT19A/FM7i, ActGFP; y^1^ w* mldr^C^ FRT19A/FM7i, ActGFP; y^1^ sc^*^ v^1^ sev^21^; P(TKO.GS00939)attP40; y^1^ sc* v^1^; scuTRiP^GL01079^; y^1^ w*; P(w[+mC] = tubPGAL4)LL7/TM3, P(w[+mC] = ActGFP)JMR2, Ser^1^; y^1^ w*; P(w[+mC] = Act5C-GAL4) 17bFO1/TM3, P(w[+mC] = ActGFP)JMR2, Ser; y^1^ M(vas-Cas9)ZH-2A w^1118^/FM7c. The following stocks were obtained from the Vienna *Drosophila* Research Center: y w^1118^; mldr^KK108043^; w^1118^; rswl^GD12447^; w^1118^; P(GD10927) v43752. The following stock was obtained from the *Drosophila* Research and Screening Center—Biomedical Technology Research Resource, Harvard Medical School: y^1^ sc* v^1^ P(nos-Cas9) attp2.

### 4.2. Transgenic Flies and Crosses

*rswl* and *scu* knockout flies were generated using CRISPR/Cas9. For *rswl*, two guide RNAs complementary to the 5′- and 3′-ends were used. The guide RNAs were commercially cloned in pU6-Bbsl-chiRNA vector (Genewiz, Plainfield, NJ, USA) expressing the chiRNA under the control of the *Drosophila* snRNA:U6:96Ab promoter [55] (Addgene, Watertown, MA, USA). 250 ng of each guide RNA expressing plasmid was commercially injected into *y^1^* M(*vas-Cas9*)*ZH-2A w^1118^*/*FM7c* stock, stably expressing Cas9 under the control of the germline-specific vasa promoter (BestGene Inc., Chino Hills, CA, USA). Positive second filial (F2) flies were confirmed by sequencing. For *scu* CRISPR knockouts, transgenic flies stably expressing guide complementary to the 5′-end of the protein-coding region (generated by Harvard Medical School, DRSC/TRiP Functional Genomics Resources) were crossed with flies stably expressing Cas9 under the control of the germline specific *nos* promoter. Positive F2 flies were confirmed by sequencing. 

### 4.3. Roswell and Scully Antibody Generation

The *Drosophila* ESTs *rswl* (LD44982) and *scu* (IP05285) (*Drosophila* Genomics Resource Center, Bloomington, IN, USA) were cloned into a p-DEST vector and transformed into BL21(DE3) cells. Transformed clones were grown at 37 °C for 5 h and induced with 0.4 mM IPTG at 18 °C overnight. The cells were harvested and lysed with sonication in binding buffer (5 mM imidazole, 500 mM NaCl, 20 mM Tris pH 7.9, and 6 M guanidine hydrochloride). Proteins were isolated and purified using HisPur Ni-NTA resin column (Fisher Scientific, Waltham, MA) as per the manufacturer’s recommended protocol. The proteins were washed in wash buffer (30 mM imidazole, 500 mM NaCl, 20 mM Tris pH 7.9, and 6 M guanidine hydrochloride) and eluted in elution buffer (300 mM imidazole, 500 mM NaCl, 20 mM Tris pH 7.9, and 6 M urea). The protein samples were pooled, concentrated, and their purity was checked by SDS-PAGE. Full-length protein samples were then sent for commercial injections into guinea pigs (Rswl) or rats (Scu) (Covance Research Products, Inc., Denver, PA, USA). 

### 4.4. Pupation and Eclosion Rates

Pupation and eclosion rates were calculated as previously described [29]. In short, twenty non-GFP (mutant) first instar larvae were transferred into vials at room temperature. The number of pupae was counted every 24 h. The number of eclosed adults was counted each day after the onset of eclosion. For each genotype, the experiment was performed in triplicates. The fly food was kept moist using water drops to avoid drying. The average and standard deviation calculations were done using GraphPad PRISM and Microsoft Excel. 

### 4.5. Immunofluorescence

Immunofluorescence was performed on samples as previously described [29]. In short, larval brains and ovaries from well-fed adult females were dissected in Grace’s insect medium (BioWhittaker, Lonza, Cologne, Germany) and fixed in Grace’s medium containing 4% paraformaldehyde and 20 mM formic acid (Sigma, St. Louis, MO, USA). Antibody staining was performed overnight at 4 °C in antibody wash buffer (1X PBS, 0.1% TritonX-100, 1% BSA). The following primary antibodies were used: guinea pig anti-Rswl (1:500) (this work), rat anti-Scu (1:500) (this work), mouse anti-ATP5A (15H4C4) (1:750) (Cat# ab14748) (Abcam, Cambridge, UK). Following primary antibody labeling the samples were washed 3x in antibody wash buffer and labeled with secondary antibodies overnight at 4 °C. The following secondary antibodies were used: goat anti-guinea pig IgG Alexa 488 (Cat# A-11073), goat anti-mouse IgG2b Alexa 568 (Cat# A-21144), goat anti-mouse IgG Alexa 488 (Cat# A-11001) (1:500) (Molecular Probes, Invitrogen, Carlsbad, CA, USA), and donkey anti-rat IgG Cy3 (1:500) (Cat# 712-165-153) (Jackson Labs, West Grove, PA, USA). After washing 3x in antibody wash buffer, DAPI was added to the samples for five minutes before treating with Vectashield (Vector Laboratories, Inc., Burlingame, CA, USA) overnight at 4 °C. For labeling actin in the neuroblast cells, rhodamine-phalloidin (1:1000) (Cat# A12380) (Molecular Probes, Invitrogen, Carlsbad, CA, USA) was used along with primary antibody. Images were collected using Zeiss 700 confocal microscope 63× Plan Apo NA 1.4 lens.

### 4.6. ATP Assay

ATP assays were performed as previously described [29]. In short, age-matched wild-type and mutant larvae were collected and homogenized in ATP extraction buffer (100 mM Tris-Cl, pH 8.0, 4 mM EDTA, pH 8.0, and 6 M guanidine hydrochloride) and used for both ATP and bicinchoninic acid (BCA) assay. ATP concentrations were determined using ATP determination Kit (Invitrogen, Thermo Fischer Scientific, Waltham, MA, USA) as per the manufacturer’s protocol. The luciferase activity was measured in 96-well format using a CLARIOstar plate reader (BMG Labtech Inc., Cary, NC, USA). Protein concentrations were determined using a BCA assay. 5 µL of diluted samples (1:50) were mixed with 100 µL of BCA reagent (Pierce BCA protein assay Kit, Thermo Fischer Scientific, Waltham, MA, USA), and protein concentrations were measured on a CLARIOstar plate reader (BMG Labtech Inc., Cary, NC, USA). Results were averaged over three (Rswl) or five (Scu) technical replicates and represented as ATP concentrations normalized to the protein concentrations.

### 4.7. Western Blots

Western blots were performed as previously described [29]. For determining the protein levels in mutants and wild-type, homogenized first instar larval extracts collected in 1× PBS were used. For the antibody specificity experiment, mitochondrial pellet and cytoplasmic extract were isolated as per the manufacturer’s instructions (Mitochondria Isolation Kit for Cultured Cells, Thermo Scientific, Waltham, MA, USA). For the maternal deposition experiment, homogenized extracts of either stage 14 eggs, dissected from the ovaries of well-fed females, or mutant embryos collected 16 h after egg laying were collected in 1× PBS. The antibodies used for Western blots were guinea pig anti-Rswl (1:3333) (this work), rat anti-Scu (1:3333) (this work), rabbit anti-Mldr (1:3333) [29], mouse anti-ATP5A (15H4C4) (1:100,000) (Cat# ab1474) (Abcam, Cambridge, UK), and mouse anti-α-tubulin (1:3333) (Cat# AA4.3) (Developmental Studies Hybridoma Bank, University of Iowa, Iowa City, IA, USA). Antibodies used for loading controls were obtained in parallel.

### 4.8. qPCR

RNA was isolated from ~100 age-matched one-day-old larvae using mirVana miRNA Isolation Kit (Invitrogen, Thermo Fisher Scientific, Waltham, MA) as per the manufacturer’s recommended protocol. 250 ng of RNA was reverse transcribed using a High-Capacity cDNA Reverse Transcription kit (Applied Biosystems, Foster City, CA, USA) following the manufacturer’s recommendation. For gene expression analysis, quantitative PCR was performed using TaqMan Gene Expression Master Mix (Applied Biosystems, Foster City, CA, USA) in a 10 ul reaction with 1 µL undiluted cDNA and one of the following TaqMan probes: Dm01827021_s1 (rswl), Dm02362013_s1 (scu), Dm01833186_g1(mldr), Dm02151827_g1 (RPL32) (endogenous control) and Dm01812859_g1 (RNase Z) (Thermo Fisher Scientific, Waltham, MA). For junction quantitative PCR, 1 µL of cDNA diluted to 1:10 was used in a 10 µL reaction with Platinum SYBR Green qPCR SuperMix-UDG (Invitrogen, Carlsbad, CA) and specific primers (Supplemental Table S1) adopted from Clemente et al. [56]. qPCR was performed in triplicates for each genotype, and the delta delta Ct (ddCt) calculations were performed using reference RNA for RpL32 (gene qPCR) or 18S (junction qPCR) as endogenous controls and *y w*, as a wild-type control. Shown below are the general calculations for junction qPCR:

dCt junction = Ct(junction)—Ct(18S reference) [within each sample]

ddCT = dCt (mutant)—dCt (*y w* control) 

[dCt (*y w* control) = (mean Ct(junction)—mean Ct(18S reference)]

We represented the values for *y w* control on the graphs for additional ease of comparison between mutant and wild-type. For the *y w* bars, we calculated the ddCt as follows:

ddCt = dCt (*y w* control, individual dCt)—dCt (*y w* control, mean Ct(junction)—mean Ct(18S reference))

Each bar on the graph represents the average of three 2^ddCt to better represent the exponential nature of PCR. The error bars represent the standard error of mean (S.E.M.) calculated with the 2^ddCt values. *p*-values were calculated using an unpaired *t*-test, one-tailed, and used to compare each individual mutant vs. *y w* (wild-type). The hypothesis we were testing was that there was a difference between wild-type and mutant in the amount of available intact mtRNA across each junction that can give rise to PCR product (dCt) and our null hypothesis was that the dCt values were equal between wild-type and mutants. Since dCt values have a normal distribution due to the nature of the assay, we used a *t*-test to compare the significance between ddCt of product from wild-type and ddCt of product from mutant. We used one-tailed for the junction analysis because we were measuring less efficient transcript processing and thus increases in PCR product across the junction compared to wild-type and were not testing more efficient transcript processing with concomitant decreases in PCR product. Since we were testing for positive and negative changes for the qPCR measuring mtRNA for genes, we used a two-tailed analysis. The dCt calculations were performed in Microsoft Word Excel. The S.E.M. and *p*-values were calculated using GraphPad PRISM. The graphs were plotted using GraphPad PRISM.

## Supplementary Materials

The following are available online at https://www.mdpi.com/article/10.3390/ijms22116066/s1, Figure S1: *scu* and *rswl* CRISPR/Cas9 generated alleles. Figure S2: Additional analysis of *scu*, *rswl*, and *mldr* alleles, Figure S3: Rswl and Scu colocalize with mitochondria, Figure S4: mtRNase P is maternally loaded, Figure S5: Northern dot blot analysis of mt:tRNA^Asn^ and COIII levels, Table S1. List of qPCR primers, Supplemental Materials and Methods.
