# Supplementary material for: Loss of Individual Mitochondrial Ribonuclease P Complex Proteins Differentially Affects Mitochondrial tRNA Processing In Vivo
Source: Int J Mol Sci. 2021 Jun 4;22(11):6066. doi: 10.3390/ijms22116066 (PMC8200052; doi:10.3390/ijms22116066)
Supplement: Supplementary file 1 [file ijms-22-06066-s001.zip › ijms-1233253-supplementary.pdf]

Figure S1

A

Scu WT: 1 **M I K N A V S L V T G G A S G L G R A T** 20
1 ATGATCAAGAACGCCGTTTCCCTGGTGACCGGAGGAGCCTCTGGACTGGGCCGCGCCACC 60

Scu^{KO7}: 1 **M I K N A V S L V** S R R S L W T G P R H...54aa..*
1 ATGATCAAGAACGCCGTTTCCCTGGTCTCCCGGAGGAGCCTCTGGACTGGGCCGCGCCAC

Scu^{KO11}: 1 **M I K N A V S L V T** E P L D W A A P P...10aa..*
1 ATGATCAAGAACGCCGTTTCCCTGGTGAC----GGAGCCTCTGGACTGGGCCGCGCCACC

B

Rswl WT: 421 R Q R R E G L E F Q L D T W A T G K Q K K K Q R Q N *
1261 AGGCAGCGACGGGAGGGCTTGGAGTTCCAACTGGACACTTGGGCAACGGGGAAGCAAAAGAAAAAGCAGAGACAAAATTAA

$Rswl^{2-3}$: 421 R Q R R E G L E F Q L D N G E A K E K A E T K L K...23aa..*
1261 AGGCAGCGACGGGAGGGCTTGGAGTTCCAACTGGA--------CAACGGGGAAGCAAAAGAAAAAGCAGAGACAAAATTAA

$Rswl^{11-10}$: 421 R Q R R E G L E F Q L D G E A K E K A E T K L K...23aa..*
1261 AGGCAGCGACGGGAGGGCTTGGAGTTCCAACTGG-----------ACGGGGAAGCAAAAGAAAAAGCAGAGACAAAATTAA

Figure S2

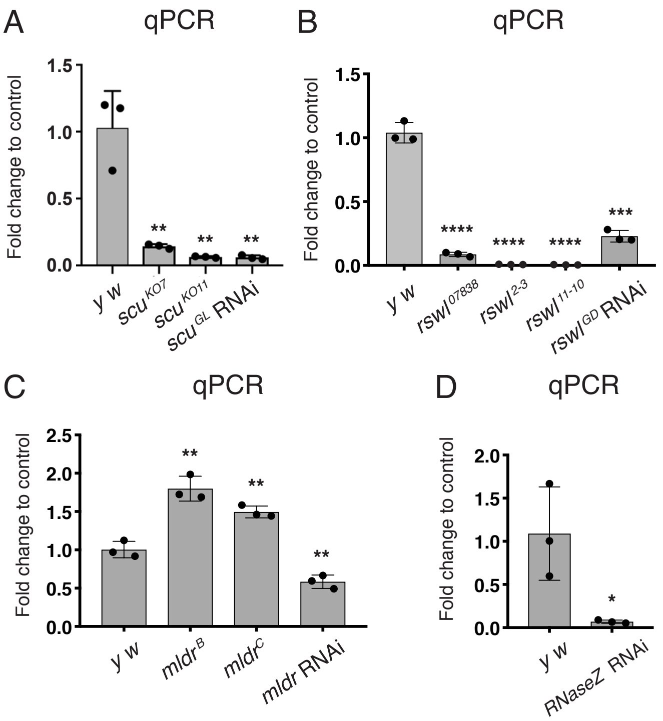
A
qPCR
Fold change to control
1.5
1.0
0.5
0.0
**
**
**
y w
scu KO7
scu KO11
scu GL RNAi
B
qPCR
Fold change to control
1.5
1.0
0.5
0.0
****
****
****
***
y w
rswl 07838
rswl 2-3
rswl 11-10
rswl GD RNAi
C
qPCR
Fold change to control
2.5
2.0
1.5
1.0
0.5
0.0
**
**
**
y w
mldr B
mldr C
mldr RNAi
D
qPCR
Fold change to control
2.0
1.5
1.0
0.5
0.0
*
y w
RNaseZ RNAi

Figure S3

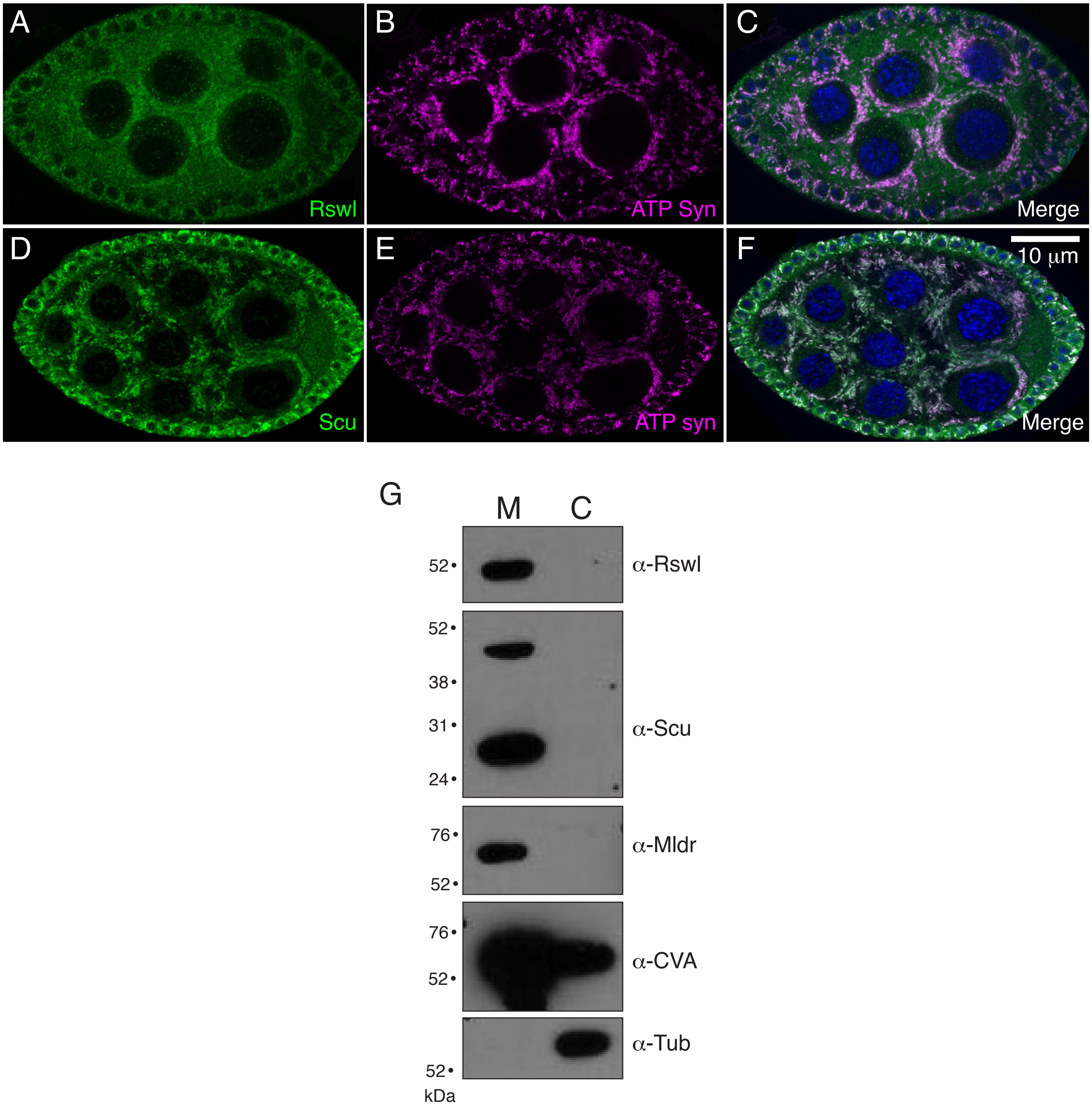
A
Rswl
B
ATP Syn
C
Merge
D
Scu
E
ATP syn
F
10 μm
Merge
G
M
C
52
α-Rswl
52
38
31
24
α-Scu
76
52
α-Mldr
76
52
α-CVA
α-Tub
52
kDa

Figure S4

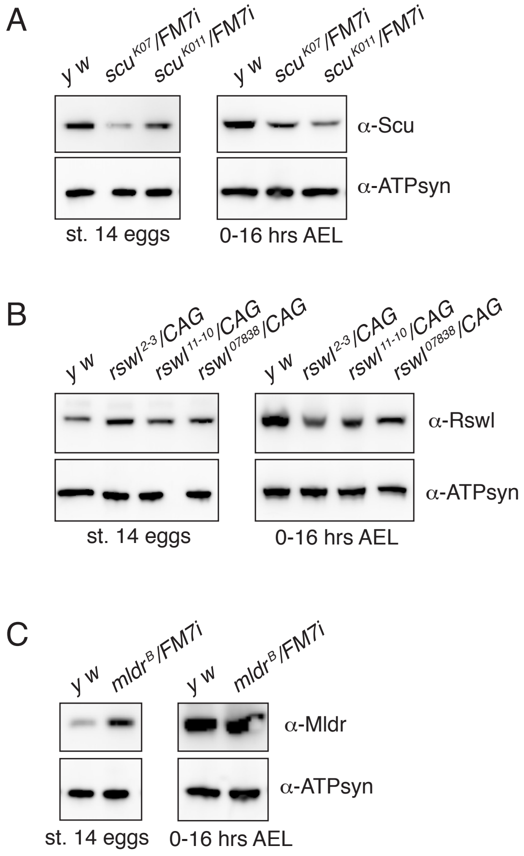
A
y w
scuK07/FM7i
scuK011/FM7i
y w
scuK07/FM7i
scuK011/FM7i
α-Scu
α-ATPsyn
st. 14 eggs
0-16 hrs AEL
B
y w
rswl2-3/CAG
rswl11-10/CAG
rswl07838/CAG
y w
rswl2-3/CAG
rswl11-10/CAG
rswl07838/CAG
α-Rswl
α-ATPsyn
st. 14 eggs
0-16 hrs AEL
C
y w
mldrB/FM7i
y w
mldrB/FM7i
α-Mldr
α-ATPsyn
st. 14 eggs
0-16 hrs AEL

Figure S5

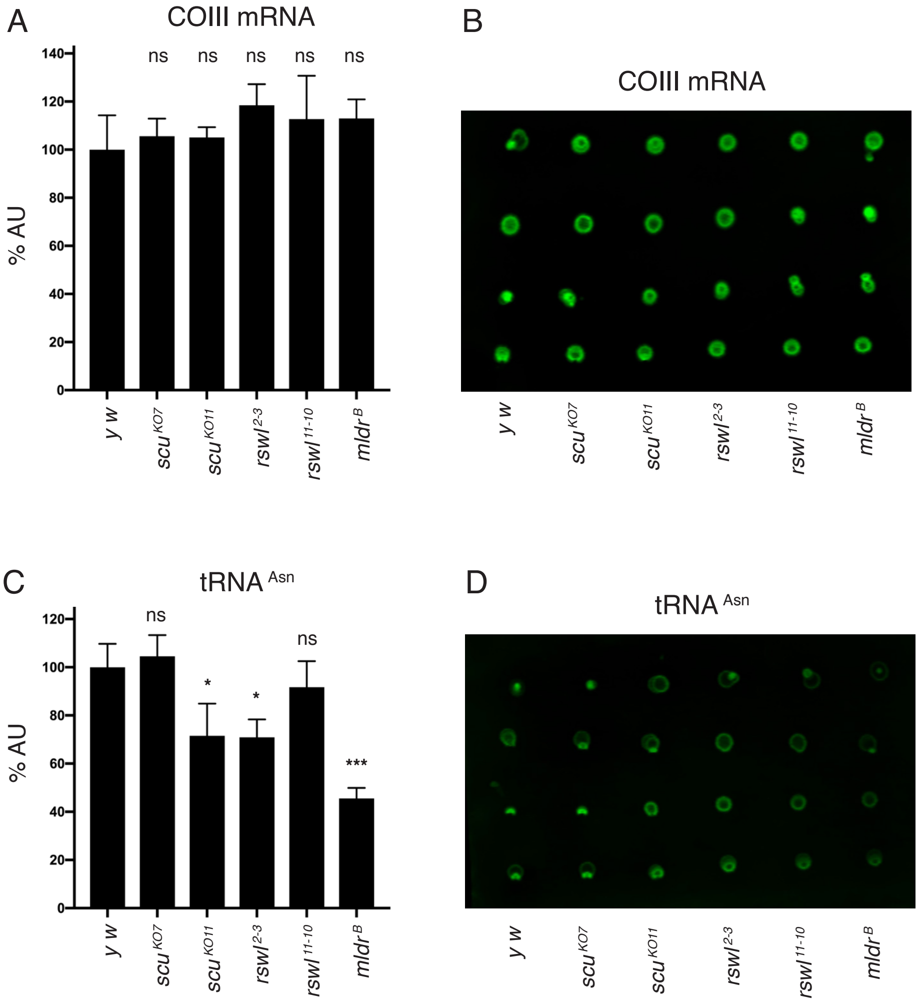
A
COIII mRNA
ns ns ns ns ns
% AU
0
20
40
60
80
100
120
140
y w
scu KO7
scu KO11
rswl 2-3
rswl 11-10
mldr B
B
COIII mRNA
y w
scu KO7
scu KO11
rswl 2-3
rswl 11-10
mldr B
C
tRNA Asn
ns
*
*
ns
***
% AU
0
20
40
60
80
100
120
y w
scu KO7
scu KO11
rswl 2-3
rswl 11-10
mldr B
D
tRNA Asn
y w
scu KO7
scu KO11
rswl 2-3
rswl 11-10
mldr B

Table S1

Name	Sequence
nc RNA - 5' tRNA Met F	TGGTGTATGATGCACAAAAGTTT
nc RNA - 5' tRNA Met R	AACCTTTATAAATGGGGTATGAACC
COIII - 5’ tRNA Gly F	TCACAATTTACTGATGAGGAGGA
COIII - 5’ tRNA Gly R	AATAGACCTTATGATTGGAAGTCAA
3’ tRNA Arg - 5’ tRNA Asn	GAAGCGATTGATTGCAATTAGTTTC
3’ tRNA Arg - 5’ tRNA Asn	CAATTATATCATTAACAGTGATATGCCTC
lr RNA - 5’ tRNA Leu(CUN	TCTAAATTTATTGCACTAATCTGCC
lr RNA - 5’ tRNA Leu(CUN	GTACGAAAGGACCAAATATCAAAA
ATP6 - 5’ COIII F	GAATCAGCTGTAGCTATAATTCAATC
ATP6 - 5’ COIII R	GTTAATGGTCATGGACTATAATCCAC
nc RNA - 5’ COI F	ATCTATCGCCTAAACTTCAGCC
nc RNA - 5’ COI R	TTTGTAGAAAATAATCATTGTCGCG
nc RNA - 5’ ND6 F	GAAAAGAAATTTCTTTTTCATTAATCCCC
nc RNA - 5’ ND6 R	GCTAATGGATGAATTATATTTAGAAAAATAATGG
ND6 - 5’ Cytb F	ATTGTAAAAATTACAAAATTATTTAAAGGACC
ND6 - 5’ Cytb R	CATTATTGGCAATTTTAAATAGAGGATGG
nc RNA - 5’ ND4L F	CGATTAGAAACAAAACAAAATAGCCC
nc RNA - 5’ ND4L R	TTTTGATTTACAAGACCAATGTT
tRNA Ile 3’ - nc RNA F	AGTTTTCTGCATTCATTGACTGA
tRNA Ile 3’ - nc RNA R	AACTTTTGTGCATCATACACCAA
tRNA Leu(CUN) 3’ - ND1	CAAATAATTAATAACAAACTACCAATTAATGAC
tRNA Leu(CUN) 3’ - ND1	TTAGTGCAATAAATTTAGAATTTATATATGTG
18S F	TGGTCTTGTACCGACGACAG
18S R	GCTGCCTTCCTTAGATGTGG
tRNA Asn_int F	TAATTGAAGCCAAAAAGAGGCATATC
tRNA Asn_int R	CTTAATTGGAATTTAAAATTCAATTATATCATTAAC
tRNA Met_int F	AAAAAGATAAGCTAATTAAGCTACTGG
tRNA Met_int R	TAAAAAGAAAAGGATTATAACCTTTATAAATGG
COIII_int F	CTGTATATCAATGATGACGAGATGTATC
COIII_int R	CTCATCGTAAACCAATAGTTACTGC
ND2_int F	GGACATTAATTACAGTTACATCTAATTC
ND2_int R	CTAAACCTATTCAAGCTCCTAACC
